# The possible importance of the antioxidants and oxidative stress metabolism in the emerging monkeypox disease: An opinion paper

**DOI:** 10.3389/fpubh.2022.1001666

**Published:** 2022-10-20

**Authors:** Duygu Aydemir, Nuriye Nuray Ulusu

**Affiliations:** ^1^Department of Medical Biochemistry, School of Medicine, Koc University, Istanbul, Turkey; ^2^Koc University Research Center for Translational Medicine (KUTTAM), Istanbul, Turkey

**Keywords:** antioxidant molecules, monkeypox, MPXV infection, oxidative stress, immune response, antioxidant enzymes

## Introduction

The world has been struggling with a major public health problem since December 2019: an infectious disease caused by a novel coronavirus called severe acute respiratory syndrome coronavirus 2 (SARS-CoV-2). Despite vaccination, people are still infected and die because of COVID-19 since the virus mutates very quickly (1, 2). While the world is struggling with the COVID-19 pandemic, a new virus called Monkeypox (MPXV) alerts scientists about whether a new pandemic will arise. Monkeypox is a zoonotic, neglected, and emerging disease caused by the MPXV belonging to the *Orthopoxvirus* genus of the *Poxviridae* family. MPXV was first identified in Macaca irus wild monkeys in 1958 in Denmark; it was the first time specified in humans in 1970 in the Democratic Republic of the Congo in a 9-month-old boy (3, 4). However, several rodent species were also reported as reservoirs of this virus (5). Monkeypox disease has been reported as an emerging outbreak affecting 43 countries with 2103 confirmed cases (6).

The transmission ways of MPXV are direct contact with an infected animal or infected person *via* body fluids, using contaminated objects, and inhaling virus-containing respiratory droplets. The incubation time of the disease takes 5–21 days, where the symptoms of MPXV infection are reported as headache, fever, muscle pain, back pain, swollen lymph nodes, chills, adenopathy, maculopapular rash, especially on the palms, and exhaustion. Lesions such as macules, papules, vesicles, pustules, and scabs have been reported mainly in the palms of the hands and the soles of the feet. There is no treatment for MPXV infection; however, smallpox vaccination is considered a treatment option (7, 8). The MPXV infection begins like other viral infections with the entry of the virus into the cells and replication, leading to the immune response in the host cells, such as blocking the antiviral T-cell activation and inflammatory cytokine production. However, cellular mechanisms of MPXV infection, host cell interactions, immune responses, and destruction are not fully understood in humans despite animals (9).

## Metabolism of virally infected cells

Viral infection and replication are tightly associated with the dysregulated immune system and inflammatory response. Since humans have complicated defense mechanisms against pathogens, viruses can quickly adapt to changing conditions such as the host's immune system and drug treatments. For instance, viruses deregulate cellular signaling pathways, including oxidative stress metabolism and cell death mechanisms, to escape the host's immune system (10, 11). The crucial step for virus replication is escaping from the cellular defense mechanism of the host cell (12, 13). Viruses are disparate from all living things; they don't inherently have their metabolism. Major cytosolic and mitochondrial metabolic pathways are altered in virus-infected cells (14, 15). Specific anabolic pathways such as glycolysis, glycogenolysis, pentose phosphate pathway (PPP), lipogenesis, cholesterol synthesis, one-carbon metabolism, and various transporters such as glucose and glutamine transporters are upregulated in virally infected cells (16, 17). It has also been investigated that the Warburg effect, which can be seen in cancer cells using glucose and producing lactate under normoxia conditions, can also be in the virus metabolism (18).

## Importance of the antioxidant defense and antioxidant molecules in the viral infections

Various intrinsic and extrinsic factors regulate oxidative stress metabolism by balancing reactive oxygen species (ROS) and antioxidant capacity. Antioxidant metabolism is one of the major defense systems in many pathological conditions, including viral infections. PPP plays a vital role in antioxidant defense by regulating different enzymes. Glucose 6-phosphate dehydrogenase (G6PD) is the rate-limiting enzyme in the PPP involved in glutathione metabolism, antioxidant response, and bioenergetic and biosynthetic pathways (19–22).

The cytosolic hexokinase enzyme rapidly converts glucose to glucose-6-phosphate (G6P) to trap the glucose inside the cell by using an ATP molecule. This enzymatic reaction is not just specific to glucose; the hexokinase enzyme phosphorylates all the six-carbon sugars. After the phosphorylation of these sugar phosphates, many cellular conditions, such as hormones, energy status, infections, and all cellular signals, determine the fate of the phosphorylated molecule. It would enter breakdown or synthesis pathways according to the metabolic signals (23–30). G6PD enzyme is found in all cells and regulates the NADP^+^/NADPH ratio involved in fatty acid, cholesterol, and neurotransmitter biosynthesis. Additionally, NADPH is the essential coenzyme in detoxification reactions *via* regulation of the balance between the oxidized glutathione (GSSG)/reduced glutathione (GSH) by involving in the glutathione reductase (GR)-catalyzed enzymatic and non-enzymatic reactions (31–34).

Furthermore, the reduced form of NADPH is also vital in cytochrome p450 superfamily-catalyzed reactions, such as cytochrome p450 monooxygenases and NADPH-cytochrome P450 reductase responsible for the xenobiotic detoxification, antioxidant-defense system, and cellular redox homeostasis. Since GSH/GSSG ratio is the major biomarker for oxidative stress, preserving the GSH pool is vital to maintaining antioxidant defense in the cell (35). Virus-infected cells also affect the mitochondrial pathways due to the high demand for biosynthetic processes such as the proliferation of virions. Mitochondria is the major source of ROS and enhanced ROS induces mitochondrial dysfunction leading to impaired electron transport chain (ETC) and energy metabolism (36). However, NADPH also protects mitochondria stress *via* a mitochondrial membrane from the effects of ROS *via* NADPH-dependent antioxidant enzymes (37). Human viral diseases, including COVID-19, increase the production of ROS and impair antioxidant mechanisms leading to the impairment of the immune system (38). On the other hand, virus-induced immune response contributes to oxidative stress as well, where oxidative stress increases inflammation, leading to enhanced oxidative stress as a vicious cycle (39). Danger signals trigger the immune system through pattern recognition receptors (PRRs) belonging to the Toll-like (TLRs) and the NOD-like (NLRs) families, where oxidative stress involves in these processes at several levels, including the release of danger molecules, activation by PRRs, and their downstream pathways (40). All viral infections cause redox imbalance in the host; for instance, prototypic poxvirus vaccinia virus (VACV) enhances ROS production at the side of the infection to promote viral replication. Additionally, high levels of ROS are required for VACV infection (41).

Antioxidant administration has been reported to ameliorate virus-induced side effects or to reduce viral replication yield, according to various studies. For instance, N-acetyl-L-cysteine (NAC) inhibits pro-inflammatory mediators in the alveolar cells infected with influenza virus A and B and with the respiratory syncytial virus (RSV) (42). The antioxidant molecule butylated hydroxyanisole (BHA) treatment ameliorates RSV-induced lung inflammation (43). Terameprocol (TMP) is a methylated derivative of nordihydroguaiaretic acid, which is a phenolic antioxidant derived from creosote bush. TMP showed antiviral and anti-inflammatory effects *via* potently inhibiting the growth of both cowpox virus and vaccinia virus *in vitro*, where TMP treatment effectively reduced the infectious virus yield (44). On the other hand, resveratrol altered genome replication and post-replicative gene expression of MXPV (45). Resveratrol (RV) is a natural polyphenol non-flavonoid compound found in grapes, berries, and several other plants. RV is accepted as one of the powerful polyphenols with many positive effects on metabolism and health and significantly reduces the replication of MPXV (46, 47). No studies reveal the antioxidant's impact on the MPXV infection in humans since monkeypox is an emerging disease worldwide. Thus, the possible effect of the antioxidants on the MPXV infection in humans can be investigated to develop antioxidant-based therapeutic approaches to ease the severe symptoms.

## Conclusion

All viruses depend entirely on the host's cell cellular metabolism, and every virus family has different molecular machinery to enter, using the host cells' energy and metabolic pathways multiplication and all steps in viral infection. However, we need novel studies to increase our knowledge on virus and virus-infected host cell metabolism, especially during the pandemic and the Monkeypox outbreak. Since antioxidants can reduce MPXV replication *in vitro*, according to the studies, antioxidant molecules can be investigated to develop therapeutic approaches or to ease the symptoms of MPXV infection.

## Author contributions

DA and NU are responsible for the conceptualization and writing the manuscript. All authors contributed to the article and approved the submitted version.

## Conflict of interest

The authors declare that the research was conducted in the absence of any commercial or financial relationships that could be construed as a potential conflict of interest.

## Publisher's note

All claims expressed in this article are solely those of the authors and do not necessarily represent those of their affiliated organizations, or those of the publisher, the editors and the reviewers. Any product that may be evaluated in this article, or claim that may be made by its manufacturer, is not guaranteed or endorsed by the publisher.

## Author disclaimer

The content is solely the responsibility of the authors and does not necessarily represent the official views of the Presidency of Strategy and Budget.

## References

[1] Aydemir D Ulusu NN. Correspondence: angiotensin-converting enzyme 2 coated nanoparticles containing respiratory masks, chewing gums and nasal filters may be used for protection against COVID-19 infection. Travel Med Infect Dis. (2020) 37:101697. 10.1016/j.tmaid.2020.101697 32335209 PMC7195061

[2] Harapan H Itoh N Yufika A Winardi W Keam S Te H. Coronavirus disease 2019 (COVID-19): a literature review. J Infect Public Health. (2020) 13:667–73. 10.1016/j.jiph.2020.03.019 32340833 PMC7142680

[3] Parker S Buller RM. A review of experimental and natural infections of animals with monkeypox virus between 1958 and 2012. Fut Virol. (2013) 8:129–57. 10.2217/fvl.12.130 23626656 PMC3635111

[4] Alakunle E Moens U Nchinda G Okeke MI. Monkeypox virus in nigeria: infection biology, epidemiology, and evolution. Viruses. (2020) 12:1257. 10.3390/v12111257 33167496 PMC7694534

[5] Silva NIO de Oliveira JS Kroon EG Trindade GS Drumond BP. Here, there, and everywhere: the wide host range and geographic distribution of zoonotic orthopoxviruses. Viruses. (2020) 13:43. 10.3390/v13010043 33396609 PMC7823380

[6] WHO. Multi-Country Monkeypox Outbreak: Situation Update. WHO. Available online at: https://www.who.int/emergencies/disease-outbreak-news/item/2022-DON393

[7] Cann JA Jahrling PB Hensley LE Wahl-Jensen V. Comparative pathology of smallpox and monkeypox in man and macaques. J Comp Pathol. (2013) 148:6–21. 10.1016/j.jcpa.2012.06.007 22884034 PMC3498598

[8] Peter OJ Kumar S Kumari N Oguntolu FA Oshinubi K Musa R. Transmission dynamics of monkeypox virus: a mathematical modelling approach. Model Earth Syst Environ. (2021) 8:3423–34. 10.1007/s40808-021-01313-2 34667829 PMC8516625

[9] Tortorella D Gewurz BE Furman MH Schust DJ Ploegh HL. Viral subversion of the immune system. Annu Rev Immunol. (2000) 18:861–926. 10.1146/annurev.immunol.18.1.861 10837078

[10] Hanada S Pirzadeh M Carver KY Deng JC. Respiratory viral infection-induced microbiome alterations and secondary bacterial pneumonia. Front Immunol. (2018) 9:2640. 10.3389/fimmu.2018.02640 30505304 PMC6250824

[11] Pratheek BM Saha S Maiti PK Chattopadhyay S Chattopadhyay S. Immune regulation and evasion of mammalian host cell immunity during viral infection. Indian J Virol. (2013) 24:1–15. 10.1007/s13337-013-0130-7 24426252 PMC3650184

[12] de Beeck AO Caillet-Fauquet P. Viruses and the cell cycle. In: Progress in Cell Cycle Research. Boston, MA: Springer US (1997). p. 1–19. 10.1007/978-1-4615-5371-7_1 9552402

[13] Meylan E Tschopp J. Toll-Like receptors and RNA helicases: two parallel ways to trigger antiviral responses. Mol Cell. (2006) 22:561–9. 10.1016/j.molcel.2006.05.012 16762830

[14] Morrison AJ. Cancer cell metabolism connects epigenetic modifications to transcriptional regulation. FEBS J. (2022) 289:1302–14. 10.1111/febs.16032 34036737 PMC8613311

[15] Gusev E Zhuravleva Y. Inflammation: a new look at an old problem. Int J Mol Sci. (2022) 23:4596. 10.3390/ijms23094596 35562986 PMC9100490

[16] Perła-Kaján J Jakubowski H. COVID-19 and one-carbon metabolism. Int J Mol Sci. (2022) 23:4181. 10.3390/ijms23084181 35456998 PMC9026976

[17] Mayer KA Stöckl J Zlabinger GJ Gualdoni GA. Hijacking the supplies: metabolism as a novel facet of virus-host interaction. Front Immunol. (2019) 10:1533. 10.3389/fimmu.2019.01533 31333664 PMC6617997

[18] Icard P Lincet H Wu Z Coquerel A Forgez P Alifano M. The key role of Warburg effect in SARS-CoV-2 replication and associated inflammatory response. Biochimie. (2021) 180:169–77. 10.1016/j.biochi.2020.11.010 33189832 PMC7659517

[19] Aydemir D Ulusu NN. Is glucose-6-phosphate dehydrogenase enzyme deficiency a factor in Coronavirus-19 (COVID-19) infections and deaths? Pathog Glob Health. (2020) 114:109–10. 10.1080/20477724.2020.1751388 32286926 PMC7241485

[20] Aydemir D Daglioglu G Candevir A Kurtaran B Bozdogan ST Inal TC. COVID-19 may enhance risk of thrombosis and hemolysis in the G6PD deficient patients. Nucleosides Nucleotides Nucleic Acids. (2021) 40:505–17. 10.1080/15257770.2021.1897457 33719907

[21] Lee SR Roh JY Ryu J Shin HJ Hong EJ. Activation of TCA cycle restrains virus-metabolic hijacking and viral replication in mouse hepatitis virus-infected cells. Cell Biosci. (2022) 12:7. 10.1186/s13578-021-00740-z 35042550 PMC8764321

[22] Moreno-Altamirano MMB Kolstoe SE Sánchez-García FJ. Virus control of cell metabolism for replication and evasion of host immune responses. Front Cell Infect Microbiol. (2019) 9:95. 10.3389/fcimb.2019.00095 31058096 PMC6482253

[23] Aydemir D Öztaşci B Barlas N Ulusu NN. Effects of butylparaben on antioxidant enzyme activities and histopathological changes in rat tissues. Arch Indust Hyg Toxicol. (2019) 70:315–24. 10.2478/aiht-2019-70-3342 32623865

[24] Gök M Ulusu NN Tarhan N Tufan C Ozansoy G Ari N. Flaxseed protects against diabetes-induced glucotoxicity by modulating pentose phosphate pathway and glutathione-dependent enzyme activities in rats. J Diet Suppl. (2016) 13:339–51. 10.3109/19390211.2015.1036188 26317558

[25] Nóbrega-Pereira S Fernandez-Marcos PJ Brioche T Gomez-Cabrera MC Salvador-Pascual A Flores JM. G6PD protects from oxidative damage and improves healthspan in mice. Nat Commun. (2016) 7:10894. 10.1038/ncomms10894 26976705 PMC4796314

[26] Bermúdez-Muñoz JM Celaya AM Hijazo-Pechero S Wang J Serrano M Varela-Nieto I. *G6PD* overexpression protects from oxidative stress and age-related hearing loss. Aging Cell. (2020) 19:e13275. 10.1111/acel.13275 33222382 PMC7744953

[27] Dashty M. A quick look at biochemistry: carbohydrate metabolism. Clin Biochem. (2013) 46:1339–52. 10.1016/j.clinbiochem.2013.04.027 23680095

[28] Judge A Dodd MS. Metabolism. Essays Biochem. (2020) 64:607–47. 10.1042/EBC20190041 32830223 PMC7545035

[29] Tandogan B Kuruüzüm-Uz A Sengezer C Güvenalp Z Demirezer LÖ Nuray Ulusu N. *In vitro* effects of rosmarinic acid on glutathione reductase and glucose 6-phosphate dehydrogenase. Pharm Biol. (2011) 49:587–94. 10.3109/13880209.2010.533187 21554000

[30] Ulusu NN. Glucose-6-phosphate dehydrogenase deficiency and Alzheimer's disease: partners in crime? The hypothesis. Med Hypotheses. (2015) 85:219–23. 10.1016/j.mehy.2015.05.006 26004559

[31] Aydemir D Hashemkhani M Durmusoglu EG Acar HY Ulusu NN. A new substrate for glutathione reductase: glutathione coated Ag2S quantum dots. Talanta. (2019) 194:501–6. 10.1016/j.talanta.2018.10.049 30609564

[32] Ulusu NN Acan NL Turan B Tezcan EF. Inhibition of glutathione reductase by cadmium ion in some rabbit tissues and the protective role of dietary selenium. Biol Trace Elem Res. (2003) 91:151–6. 10.1385/BTER:91:2:151 12719610

[33] Ulusu NN Tandogan B. Purification and kinetic properties of glutathione reductase from bovine liver. Mol Cell Biochem. (2007) 303:45–51. 10.1007/s11010-007-9454-1 17410407

[34] Aydemir D Ulusu NN. Comment on the: molecular mechanism of CAT and SOD activity change under MPA-CdTe quantum dots induced oxidative stress in the mouse primary hepatocytes (Spectrochim Acta A Mol Biomol Spectrosc. 2019 Sep 5; 220:117104). Spectrochim Acta A Mol Biomol Spectrosc. (2020) 229:117792. 10.1016/j.saa.2019.117792 31865110

[35] Manikandan P Nagini S. Cytochrome P450 structure, function and clinical significance: a review. Curr Drug Targets. (2018) 19:38–54. 10.2174/1389450118666170125144557 28124606

[36] Combs JA Norton EB Saifudeen ZR Bentrup KHZ Katakam P v Morris CA. Human cytomegalovirus alters host cell mitochondrial function during acute infection. J Virol. (2020) 94:e01183–19. 10.1128/JVI.01183-19 31694945 PMC6955246

[37] Tarafdar A Pula G. The role of NADPH oxidases and oxidative stress in neurodegenerative disorders. Int J Mol Sci. (2018) 19:3824. 10.3390/ijms19123824 30513656 PMC6321244

[38] Hejrati A Nurzadeh M Roham M. Association of coronavirus pathogencity with the level of antioxidants and immune system. J Fam Med Prim Care. (2021) 10:609. 10.4103/jfmpc.jfmpc_1007_20 34041049 PMC8138403

[39] Aydemir D Ulusu NN. People with blood disorders can be more vulnerable during COVID-19 pandemic: a hypothesis paper. Transfus Apher Sci. (2021) 60:103080. 10.1016/j.transci.2021.103080 33608217 PMC7874911

[40] Lugrin J Rosenblatt-Velin N Parapanov R Liaudet L. The role of oxidative stress during inflammatory processes. Biol Chem. (2014) 395:203–30. 10.1515/hsz-2013-0241 24127541

[41] Bidgood SR Samolej J Novy K Collopy A Albrecht D Krause M. Poxviruses package viral redox proteins in lateral bodies and modulate the host oxidative response. PLoS Pathog. (2022) 18:e1010614. 10.1371/journal.ppat.1010614 35834477 PMC9282662

[42] Mata M Morcillo E Gimeno C Cortijo J. N-acetyl-l-cysteine (NAC) inhibit mucin synthesis and pro-inflammatory mediators in alveolar type II epithelial cells infected with influenza virus A and B and with respiratory syncytial virus (RSV). Biochem Pharmacol. (2011) 82:548–55. 10.1016/j.bcp.2011.05.014 21635874

[43] Castro SM Guerrero-Plata A Suarez-Real G Adegboyega PA Colasurdo GN Khan AM. Antioxidant treatment ameliorates respiratory syncytial virus–induced disease and lung inflammation. Am J Respir Crit Care Med. (2006) 174:1361–9. 10.1164/rccm.200603-319OC 17008643 PMC2648297

[44] Pollara JJ Laster SM Petty ITD. Inhibition of poxvirus growth by terameprocol, a methylated derivative of nordihydroguaiaretic acid. Antiviral Res. (2010) 88:287–95. 10.1016/j.antiviral.2010.09.017 20888364

[45] Abdelkhalek A Salem MZM Kordy AM Salem AZM Behiry SI. Antiviral, antifungal, and insecticidal activities of eucalyptus bark extract: HPLC analysis of polyphenolic compounds. Microb Pathog. (2020) 147:104383. 10.1016/j.micpath.2020.104383 32659315

[46] Salehi B Mishra A Nigam M Sener B Kilic M Sharifi-Rad M. Resveratrol: a double-edged sword in health benefits. Biomedicines. (2018) 6:91. 10.3390/biomedicines6030091 30205595 PMC6164842

[47] Cao S Realegeno S Pant A Satheshkumar PS Yang Z. Suppression of poxvirus replication by resveratrol. Front Microbiol. (2017) 8:2196. 10.3389/fmicb.2017.02196 29204136 PMC5698801

